# Molecular Switch Role of Akt in *Polygonatum odoratum* Lectin-Induced Apoptosis and Autophagy in Human Non-Small Cell Lung Cancer A549 Cells

**DOI:** 10.1371/journal.pone.0101526

**Published:** 2014-07-03

**Authors:** Chunyang Li, Jie Chen, Bangmin Lu, Zheng Shi, Hailian Wang, Bin Zhang, Kailiang Zhao, Wei Qi, Jinku Bao, Yi Wang

**Affiliations:** 1 School of Life Sciences and Key Laboratory of Bio-resources and Eco-environment, Ministry of Education, Sichuan University, Chengdu, China; 2 Central Laboratory of Clinical Medicine, Sichuan Academy of Medical Science & Sichuan Provincial People’s Hospital, Chengdu, China; 3 Institute of Organ Transplantation, Sichuan Academy of Medical Science & Sichuan Provincial People’s Hospital, Chengdu, China; 4 Department of Pharmacy, Sichuan Academy of Medical Science & Sichuan Provincial People’s Hospital, Chengdu, China; 5 Center for Perinatal Research, Nationwide Children’s Hospital, Columbus, Ohio, United States of America; H. Lee Moffitt Cancer Center & Research Institute, United States of America

## Abstract

*Polygonatum odoratum* lectin (POL), isolated from traditional Chinese medicine herb *(Mill.)* Druce, has drawn rising attention due to its wide biological activities. In the present study, anti-tumor effects, including apoptosis- and autophagy-inducing properties of POL, were determined by a series of cell biology methods such as MTT, cellular morphology observation, flow cytometry, immunoblotting. Herein, we found that POL could simultaneously induce apoptosis and autophagy in human non-small cell lung cancer A549 cells. POL initiated apoptosis through inhibiting Akt-NF-κB pathway, while POL triggered autophagy *via* suppressing Akt-mTOR pathway, suggesting the molecular switch role of Akt in regulating between POL-induced apoptosis and autophagy. Moreover, ROS was involved in POL-induced inhibition of Akt expression, and might therefore mediate both apoptosis and autophagy in A549 cells. In addition, POL displayed no significant cytotoxicity toward normal human embryonic lung fibroblast HELF cells. Due to the anti-tumor activities, POL might become a potent anti-cancer drug in future therapy, which might pave the way for exploring GNA-related lectins into effective drugs in cancer treatment.

## Introduction

Lectins are designated as carbohydrate-binding proteins that widely exist in animals, plants and microorganisms, and they could bind carbohydrates, agglutinate cells or precipitate polysaccharides and glycoconjugates [1]. Rapid progress has been achieved in isolation and characterization of plant lectins, and recently the classification of lectins has been emended from 7 families into 12 families [2]–[4].

Of note, cancer is associated with programmed cell death (PCD), which plays important roles in homeostasis preservation, cellular differentiation, growth control, cell defense and etc., jointly sealing the ultimate fate of cancer cells [5]. Generally, there are two main types of PCD, referring to apoptosis and autophagy. Autophagy is an evolutionarily conserved cellular mechanism for clearance of damaged or superfluous macro-complexes and organelles in eukaryotic cells, which leads to either pro-survival or pro-death effects [6]. Apoptosis, which can be regulated by numerous molecular signaling pathways, is mainly targeted for tumor suicide. Autophagy and apoptosis bear distinct morphological characteristics and physiological process, however, there still exist intricate interrelationships between them [7].

*Polygonatum odoratum* (Mill.) Druce, a typical representative of the Liliaceae family, is an important traditional Chinese herbal medicine owing to its wide varieties of biologically active compounds. *Polygonatum odoratum* lectin (POL), isolated from *Polygonatum odoratum* (Mill.) Druce, is a mannose-binding specific *Galanthus nivalis* agglutinin (GNA)-related family lectin, and exerts remarkable growth-inhibition effects against A375 cells [8]. Previous report has demonstrated that POL induced L929 cell apoptosis through both death-receptor and mitochondrial pathways, as well as amplified TNFα-induced L929 cell apoptosis [9]. However, whether POL could simultaneously induce apoptosis and autophagy in cancer cells are still in their infancy. And hitherto, only *Polygonatum cyrtonema* lectin (PCL) can simultaneously induce both apoptosis and autophagy in human melanoma A375 cells [10] and L929 cells [11]. Therefore, the apoptosis- and autophagy-inducing effects of POL needs to be explored.

## Materials and Methods

### Molecular modeling

Three-dimensional structure of POL was constructed using SWISS-MODEL server (http://swissmodel.expasy.org/) with the structure of PCL (PDB ID: 3A0E) as template [12], [13].

### Cell culture

Lung adenocarcinoma A549 cell lines were purchased from American Type Culture Collection (ATCC, Manassas, USA), while normal human embryonic lung fibroblast HELF cell lines were purchased from cell bank (Chinese Academy of Sciences, Shanghai, China). Human non-small cell lung cancer A549 cells were cultured in RPMI-1640 medium (Gibco), containing 10% FBS, 100 mg/mL streptomycin (Invitrogen), 100 U/mL penicillin (Invitrogen), and maintained at 37°C with 5% CO_2_ at a humidified atmosphere. And human embryonic lung fibroblast HELF cells, used as corresponding control group, were cultured in Dulbecco minimal essential medium (Gibco) containing the same materials.

### Reagents

POL was purified and maintained by our lab [8]. Fetal bovine serum (FBS), 3-(4,5-dimetrylthiazol-2-yl)-2,5-diphenyltetrazolium bromide (MTT), 3,3-diaminobenzidine tetrahydrochloride (DAB), monodansylcadaverine (MDC), autophagy inhibitor 3-methyladenine (3-MA), acridine orange (AO), rhodamine-123 and z-VAD-fmk were purchased from Sigma Chemical (St. Louis, MO, USA). cytochrome *c* Apoptotic WB antibody cocktail (ab110415) was obtained for MitoSciences (Eugene, Oregon, US). Small interfering RNAs (siRNAs) against human Beclin1(#6222), LC3(#6212) and control siRNA(#6568) were purchased from Cell Signaling Technologies, USA.

Following antibodies were purchased from Santa Cruz Biotech: pro-caspase3(#sc-7148), pro-caspase9(#sc-56073), Bax(#sc-493), Bid(#sc-6538), Bcl-2(#sc-492), Bcl-X_L_(#sc-8392), PARP(#sc-7150), NF-κB(#sc-109), Phospho-NF-κB (Ser536) (#sc-136548) and β-actin (#sc-47778). Besides, antibodies including Phospho-Beclin1(Ser234)(ab183335), mTOR(ab2732) and Phospho-mTOR (Ser2448) (ab109268) were from Abcam. Antibodies for LC3(mAb#12741), Akt(pan)(mAb#4685), Phospho-Akt (Thr308)(mAb#2965), Phospho-Akt (Ser473)(mAb#4060), Phospho-p70S6K (Thr389)(mAb#9234) and Phospho-4EBP1 (Thr37/46)(mAb#2855) were purchased from Cell Signaling Technologies, USA. Proteins were visualized using anti-rabbit or anti-mouse IgG conjugated with peroxidase (HRP) and 3,3-diaminobenzidine tetrahydrochloride (DAB) as the HRP substrate.

Constitutively activated Akt (Myr-Akt, Addgene plasmid 9008), NF-κB (T7-RelA, Addgene plasmid 23251), mTOR (Flag-mTOR, Addgene plasmid 26603), empty vector (pcDNA3, Addgene plasmid10792) were purchased from Addgene.

### Growth inhibition assay

A549 cells were seeded into 96-well plates and cultured for indicated time, and the cytotoxic effects of various concentration of POL were performed as previously described [14]. Absorbance at 570 nm was measured with a spectrophotometer (Model 3550 Microplate Reader, Bio-Rad).

Cell viability (%) = (OD_570_nm (drug)/OD_570 _nm(control))×100%.

### Mannose inhibition assay

MTT assay was determined as mentioned above except that lectins were preincubated at 37°C for 30 min with different types of mannoses, such as mannose α (1,3), mannose α (1,6) and mannose α (1,3∶1,6), which can completely inhibit the hemagglutinating activity at different concentrations of POL.

### Apoptosis assay

A549 and HELF cells were treated with PBS and 23 µg/mL POL for 12 h, 24 h, 36 h and 48 h. Cell morphology was imaged by phase contrast microscopy (Leica, Wetzlar, Germany) as well as fluorescence microscopy (Olympus, Tokyo, Japan) with hoechst 33342 staining. Then, total of 6×10^5^ A549 cells/well were plated in 6-well plates and incubated for 24 h, and treated with PBS or 23 µg/mL POL for another 12 h, 24 h, 36 h or 48 h incubation. Harvested cells were analyzed by flow cytometry assay as described before [15]. In order to further classify early and late apoptosis in POL-treated A549 cells, Annexin V-FITC/PI double staining assay was performed by Annexin V-fluorescein isothiocyanate (FITC) apoptosis detection kit according to the manufacturer’s instructions (BD Pharmingen, San Diego, CA, USA).

### Autophagy assay

After incubation with 23 µg/mL POL for indicated time, A549 and HELF cells were cultured with 0.05 mM MDC at 37°C for 60 min. The fluorescence intensity of cells was analyzed by flow cytometry. In addition, A549 cells incubated with PBS or 23 µg/mL POL for indicated time were stained with acridine orange (10 ng/mL) for 15 min and subjected to flow cytometry. Cells were respectively transfected with Beclin1 siRNA, LC3 siRNA and control siRNA at 33 nM using Lipofectamine 2000 (Invitrogen) according to the manufacturer’s instructions. The transfected cells were used for subsequent experiments 24 h later.

### Caspase assay

Apoptosis was assessed by measuring the activity of caspase-3 by using the caspase-3 activity kit (Beyotime Institute of Biotechnology, Haimen, China) following the manufacturer’s instructions.

### Detection of mitochondrial membrane potential

Mitochondrial membrane potential was measured using the fluorescent dye rhodamine-123 as described previously [15]. The fluorescence intensity of A549 cells interfered with 23 µg/mL POL for 12 h, 24 h, 36 h and 48 h was analyzed by flow cytometry.

### Determination of intracellular ROS

After treatment with various concentration of POL for the indicated time periods, A549 cells were incubated with 10 µM 2′,7′-dichlorofluorescein diacetate (DCFH-DA) for 60 min at 37°C. The fluorescence intensity of A549 cells was measured by flow cytometry with an excitation wavelength of 480 nm and an emission wavelength of 525 nm. Intracellular GSH content and GPX enzyme activity were measured according to the manufacturer’s instructions.

### Transfections

A549 Cells were plated in 60-mm dishes with RPMI 1640 containing 10% FBS and 1% penicillin-streptomycin reached to a density of 1×10^6^ cells/dish. Then A549 cells continuously cultured in PRMI 1640 without 10% FBS and 1% penicillin-streptomycin for another 2 h. In order to form plasmid-Lipofectamine LTX complex, plasmids were mixed with 25X diluent Lipofectamine LTX, and incubated for 20 min in room temperature. Then transfections were respectively conducted in OptiMEM reduced-serum medium (Invitrogen) containing 1 mg/mL DNA, 0.1% PLUS reagent and 0.3% Lipofectamine LTX transfection reagent, and added the medium with plasmid in 60-mm dishes for another 48 h culture of A549 cells. Finally, the transfection efficiency were detected by Western Blot analysis.

### Western blot analysis

A549 cells were treated with 23 µg/mL POL for indicated time, and subsequently both adherent as well as floating cells were collected. Western blot analysis of cell lysates was performed as previously mentioned [16], [17].

### Statistical analysis of the data

All data were expressed as mean±SEM from at least three independent experiments. Statistical comparisons were made by two-way ANOVA and one-way ANOVA with Bonferroni post hoc test. A p value≤0.05 was considered as significant.

## Results

### Molecular modeling of POL

**Figure 1:**
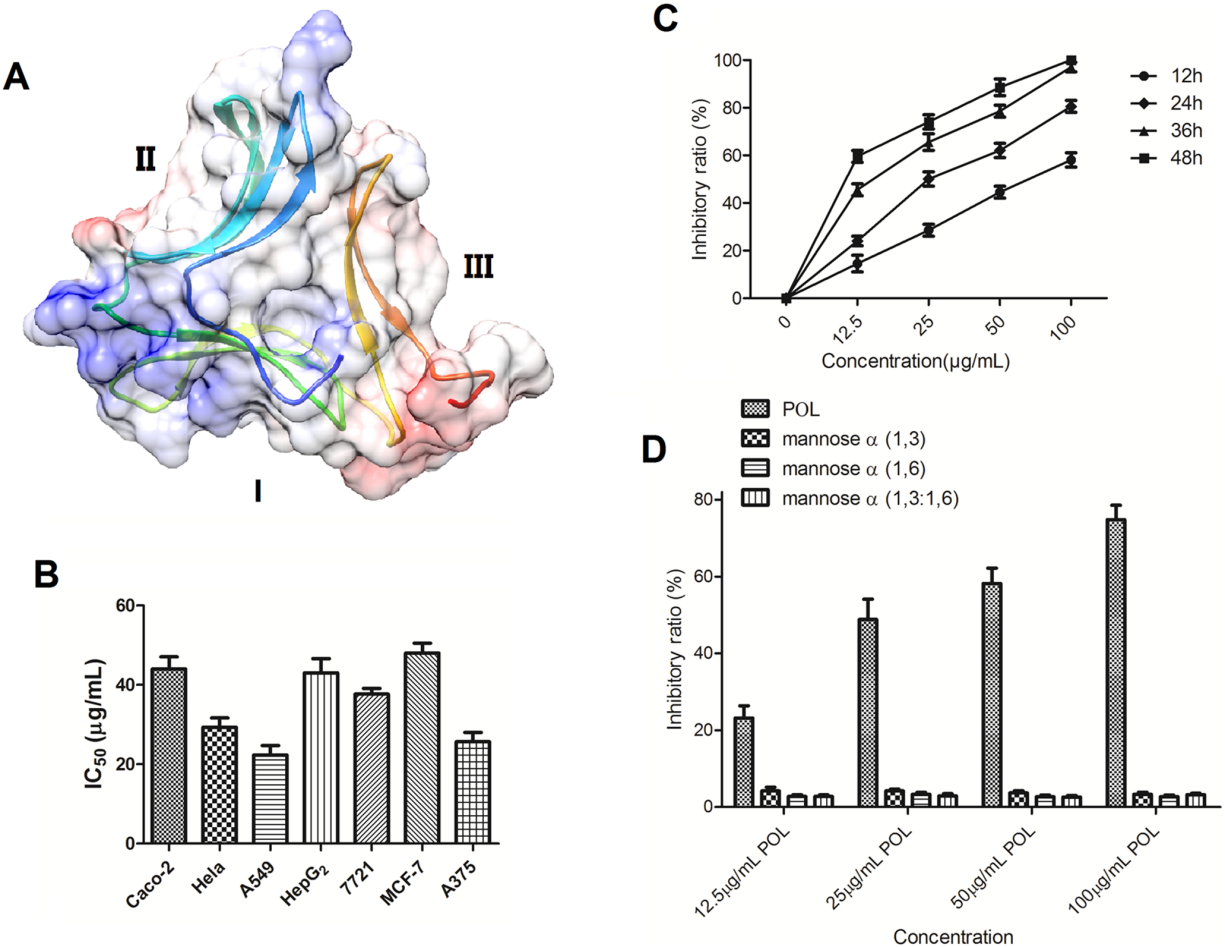
Molecular modeling, cell viability and sugar inhibition assays. (A) Molecular structure of POL monomer. (B) colon carcinoma Caco-2 cells, cervix carcinoma HeLa cells, non-small cell lung cancer A549 cells, hepatocellular carcinoma HepG_2_ and 7721 cells, breast carcinoma MCF-7 cells, melanoma A375 cells were treated with different concentrations of POL for 24 h, and the IC_50_ values were quantified by MTT assay (mean±SEM, *n* = 3). (C) A549 cells were treated with different concentration of POL, and then the cell viability was determined by the MTT assay at the indicated time (mean±SEM, *n* = 3). (D) Various concentration of POL was preincubated at 37°C for 30 min with mannose (1–3), mannose (1–6) and mannose (1–3; 1–6), and then treated with A549 cells for 24 h. The cell inhibitory ratio was measured by MTT assay (mean±SEM, *n* = 3).

Lectins from GNA-related family bear different amino acid sequence constitution, but they all exhibited similar three-dimensional structure. GNA-related family lectins bear strict mannose-binding activity, and conserved amino acid motif of ‘QXDXNXVXY’ plays important roles in mannose recognition. POL possessed three conserved ‘QXDXNXVXY’ subdomains, and therefore, POL was strict mannose-binding lectin. As shown in Fig. 1A, the three-dimensional structure of POL was built, and the monomer of POL exhibited a β-barrel structure comprising three subdomains (I, II, III), each subdomain is made up of three or four strands of antiparallel β sheet interacting by Ω loop.

### Cytotoxic effects of POL on A549 cells

The growth inhibitory effects of POL were evaluated by MTT assay on human cancer cells, including colon carcinoma Caco-2 cells, cervix carcinoma HeLa cells, non-small cell lung cancer A549 cells, hepatocellular carcinoma HepG_2_ and 7721 cells, breast carcinoma MCF-7 cells, melanoma A375 cells, and the IC_50_ values were 47 µg/mL, 30 µg/mL, 23 µg/mL, 42 µg/mL, 38 µg/mL, 50 µg/mL and 26 µg/mL, respectively (Fig. 1B). This result showed that POL was more sensitive to non-small cell lung cancer A549 cells, and hence, A549 cells were chosen for further exploration.

POL induced A549 cell death in a dose- and time-dependent manner, and 0 µg/mL to 100 µg/mL POL exerted potent inhibitory effects on the growth of A549 cells (Fig. 1C). After 24 h incubation with 23 µg/mL POL, the inhibitory rate reached nearly to 50%.

### The correlation between sugar-binding and anti-proliferative activity of POL

The mannose inhibition assay was performed to determine the relationship between sugar-binding activity of POL and its anti-proliferative property. Different concentration of POL was pre-incubated with mannose α (1,3), mannose α (1,6) and mannose α (1,3∶1,6) for 30 min, respectively. Due to the mannose-binding activity of POL, all three types of mannoses completely inhibited the hemagglutinating activity of POL. As shown in Fig. 1D, the growth inhibitory effects of various concentration of POL were lost at the corresponding level with the lose of mannose-binding activity, indicating that there may be a close link between the hemagglutinating activity and anti-proliferative property of POL.

### POL induces apoptosis in A549 cells

**Figure 2:**
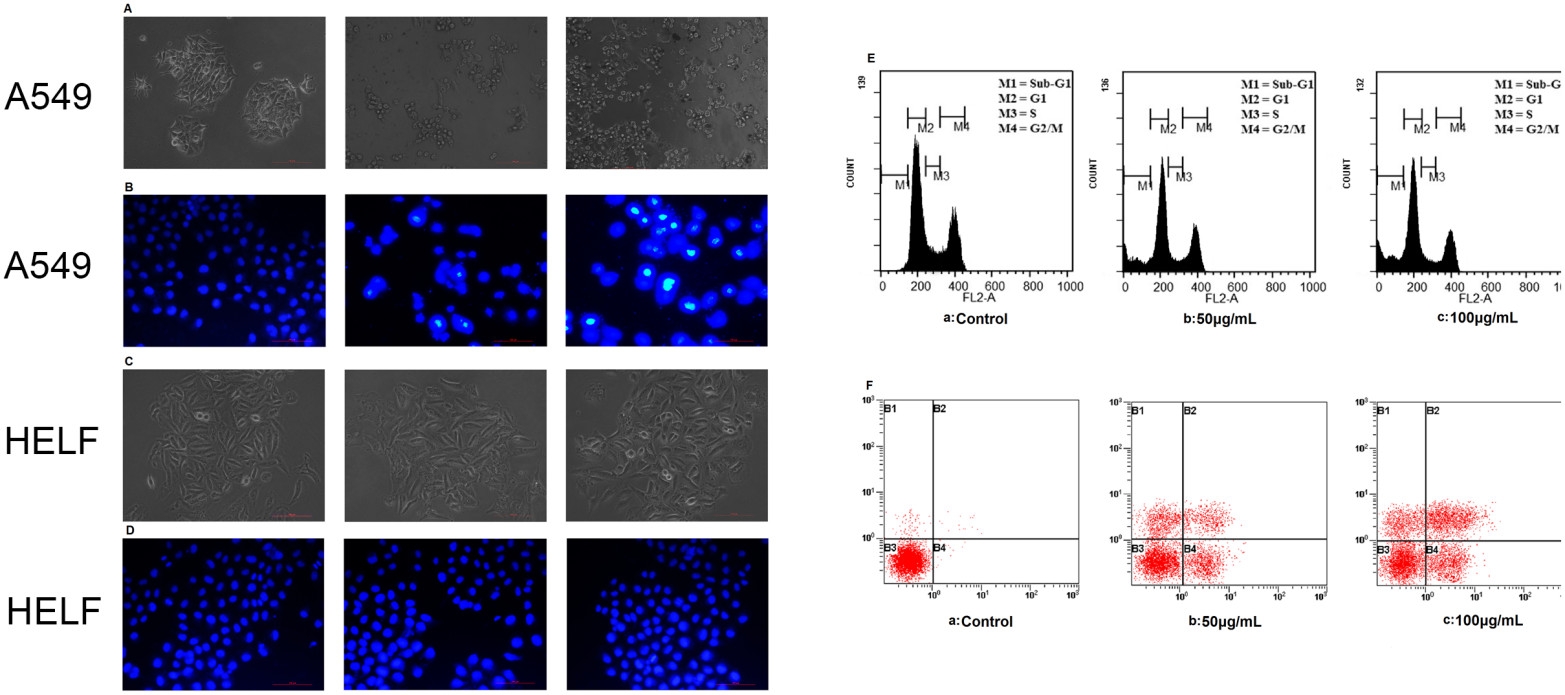
POL induces apoptosis in A549 cells. (A) A549 cells were treated with PBS (24 h) or 23 µg/mL POL for indicated time, and the morphologic varieties were detected under phase contrast microscopy and (B) fluorescent microscopy (200×). (C) After treated with HELF cells with PBS (24 h) or 23 µg/mL POL for indicated time, the morphologic varieties were detected under phase contrast microscopy and (D) fluorescent microscopy (200×). (E) Different phases percentage of A549 cells interfered with 23 µg/mL POL for 24 h or 48 h were measured by flow cytometry. Control group was treated with PBS for 24 h. (F) A549 cells were exposed to 23 µg/mL POL for 24 h or 48 h, the ratios of early apoptosis and late apoptosis were measured by flow cytometry using the Annexin V-FITC/PI double staining assay. Control group was treated with PBS for 24 h.

Marked apoptotic morphological changes as membrane blebbing, cell volume reduction and rounding, were observed in 23 µg/mL POL-treated A549 cells by phase contrast microscopy (Fig. 2A). Besides, considerable DNA fragmentation in nuclei was observed in POL-induced A549 cells, while the PBS-treated control group showed normal, round and homogeneously stained nuclei (Fig. 2B). However, 23 µg/mL POL did not result in apparent apoptotic morphology in non-cancerous HELF cells after 24 h or 48 h treatment (Fig. 2C and Fig. 2D). These results suggested that POL might selectively induce A549 cells apoptosis but could not trigger apoptosis of normal HELF cells. In addition, as shown in Fig. 2E, 23 µg/mL POL markedly led to the enhancement of Sub-G_1_ phage proportion of A549 cells in a time-dependent manner (b: 27.34±4.1%, c: 39.27±5.8%), indicating that POL induced apoptosis in A549 cells. Furthermore, Annexin V-FITC and PI double staining was performed to classify amongst alive cells (Annexin V−/PI−), early apoptotic (Annexin V+/PI−), late apoptotic (Annexin V+/PI+) and necrotic cells (Annexin V−/PI+). As shown in Fig. 2F, after 23 µg/mL POL treatment, the percentage of alive cells were gradually reduced (a: 98.25±1.3%, b: 74.63±4.7%, c: 55.71±3.9%) while the ratio of early (b: 15.34±2.6%, c: 23.75±2.8%) and late (b: 13.94±3.6%, c: 22.14±4.5%) apoptotic cells were enhanced with increasing time.

### POL triggers autophagy in A549 cells

**Figure 3:**
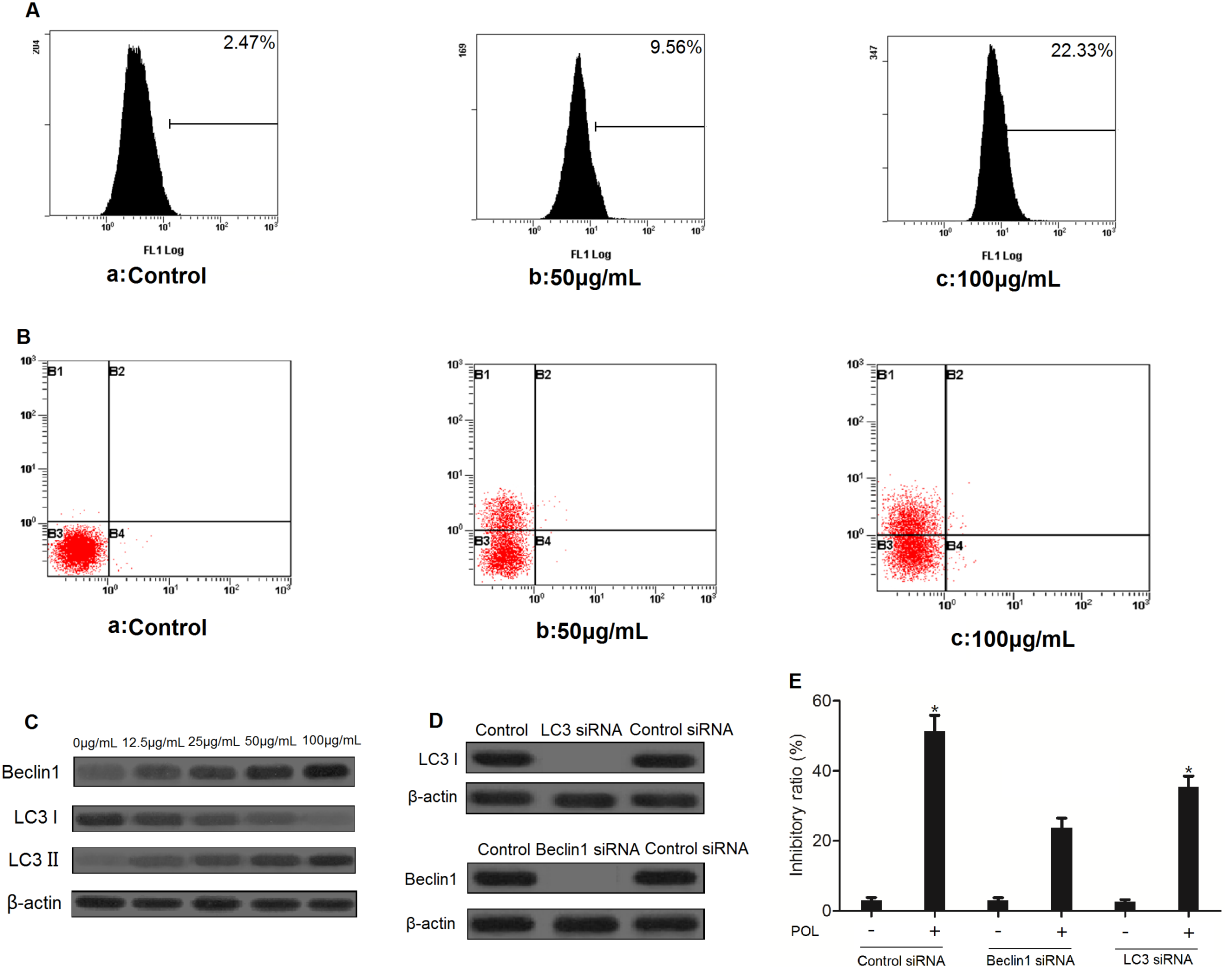
POL induces autophagy in A549 cells. A549 cells (A) and HELF cells (B) were treated with PBS (24 h) or 23 µg/mL POL for indicated time, and the MDC fluorescent intensity was analyzed by flow cytometry. (C) A549 cells were treated with PBS (24 h) or 23 µg/mL POL for indicated time, and the acridine orange fluorescent intensity was measured by flow cytometry. (D) A549 cells were treated with 23 µg/mL POL for indicated time, followed by Western blot analysis for detection of phosphorylated-Beclin1 and LC3 levels. β-actin was used as an equal loading control. (E) A549 cells were transfected with Beclin1, LC3 or control siRNA for 24 h, the Beclin1 and LC3 levels were examined by Western blot analysis. (F) A549 cells were transfected with Beclin1, LC3 or control siRNA for 24 h followed by stimulation with or without 23 µg/ml POL for 24 h, the cell growth inhibition ratio was measured by the MTT assay (mean±SEM, *n* = 3).

In order to detect the formation of autophagic vacuoles, MDC fluorescent intensity of 23 µg/mL POL-treated A549 and HELF cells for 24 h or 48 h were analyzed by flow cytometry. As shown in Fig. 3A, autophagic vacuoles were formed in A549 cells that was featured by increased MDC fluorescent intensity, indicating the presence of autophagic cell death. However, in Fig. 3B, MDC fluorescent intensity of HELF cells treated with POL was not increased neither after 24 h nor 48 h, suggesting autophagic vacuoles were not formed in POL-induced HELF cells. We could include that POL might selectively induce autophagic cell death in A549 cells but not in HELF cells. By further using acridine orange staining, the formation of acidic vesicular organelles (AVO) in POL-triggered A549 cells was demonstrated (Fig. 3C). Since Beclin1 enhancement and transformation of LC3I to LC3II were the characteristics of autophagy, the expressions of Beclin1 and LC3 in POL-induced A549 cells were detected. As shown in Fig. 3D, POL induced the time-dependent enhancement of Beclin 1 level and accumulation of the LC3-II in A549 cells. Subsequently, the expressions of Beclin1 and LC3 were knocked down by using specific siRNAs (Fig. 3E). Transfection with Beclin1 or LC3 siRNA decreased POL-induced cell growth inhibition (Fig. 3F). All these results indicated that POL induced autophagy in A549 cells.

### POL Induces caspase-dependent apoptosis in A549 cells

**Figure 4:**
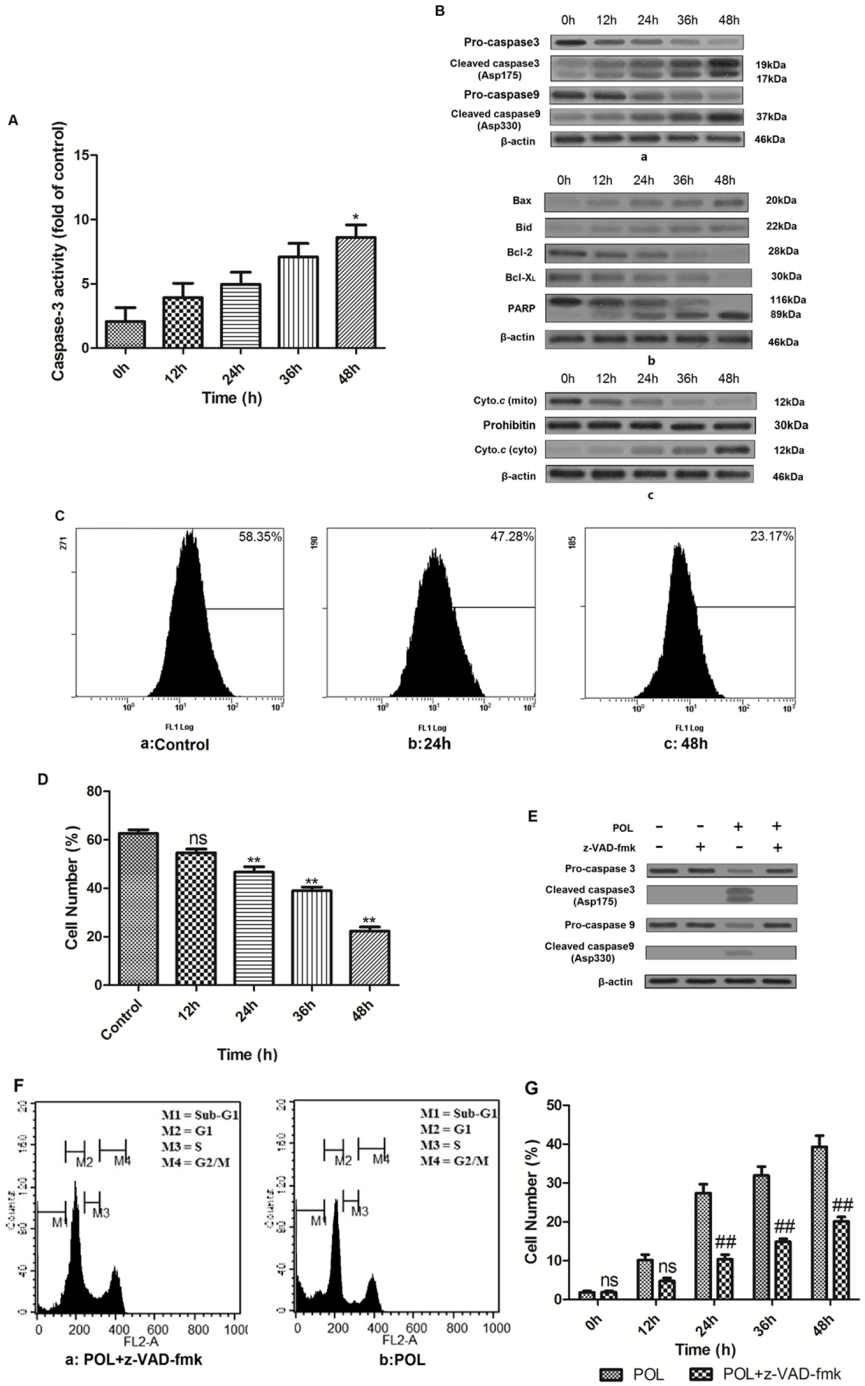
POL induces apoptosis through caspase-dependent manner. (A) Effects of 23 µg/mL POL on caspase-3 activation with increasing time (mean±SEM, *n* = 3) (*p<0.05 *vs*. 0 h group). (B) 23 µg/mL POL induced cleavage of caspase-3, caspase-9 (a). POL resulted in cleavage of PARP, up-regulation of pro-apoptotic Bax and Bid, as well as down-regulation of anti-apoptotic Bcl-2 and Bcl-X_L_ with culturing time (b). Cell lysates were fractionated to isolate cytoplasmic and crude mitochondria, and the presence of cytochrome *c* in cytosolic and mitochondrial fractions was assessed by WB analysis using the ApoTrack antibody cocktail. β-Actin was used as an cytosol equal loading control, while prohibitin was used as mitochondria loading control (c). (C) The influence of 23 µg/mL POL on the integrity of mitochondrial membranes was measured by rhodamine 123 staining, and fluorescence intensity was analyzed by flow cytometry. (D) The varieties of mitochondrial membranes potential were further presented as bar diagram (mean±SEM, *n* = 3) (**p<0.001 *vs*. Control group, ns: no significant *vs*. Control group). (E) Western blot analysis of pro-caspase 3, cleaved caspase 3, pro-caspase9 and cleaved caspase9 levels in A549 cells treated with 23 µg/mL alone or in combination with 10 µM caspase inhibitor z-VAD-fmk for 24 h. (F) A549 cells were treated with 23 µg/mL POL alone or combined with 10 µM caspase inhibitor z-VAD-fmk for 24 h and stained with propidium iodide (PI), quantitation of the PI staining data presented as the percentages of cells in SubG_1_ phase. (G) The influence of caspase on 23 µg/mL POL-induced SubG_1_ phase ratio was represented as bar diagram (mean±SEM, *n* = 3) (##p<0.001 *vs.* POL group, ns: no significant *vs.* POL group).

As shown in Fig. 4A, after autophagic inhibitor 3-MA treatment, 23 µg/mL POL significantly enhanced caspase-3 activity in a time-dependent manner, indicating POL-induced apoptosis occurs through the activation of common apoptotic executors such as caspase-3. Moreover, Western blot analyses after autophagy was inhibited showed that after treatment with 23 µg/mL POL for indicated time, caspase-3 and -9 were cleaved, since procaspase-3 and -9 were decreased while cleaved caspase-3 and -9 were increased (Fig. 4B a). Also, up-regulation of pro-apoptotic Bax and Bid proteins as well as down-regulation of anti-apoptotic Bcl-2 and Bcl-X_L_ proteins were observed after POL treatment. Besides, 23 µg/mL POL led to cleavage of PARP from an 116 kDa band to an 89 kDa fragment with increasing time (Fig. 4B b), and cytochrome *c* was released from mitochondria into cytoplasm after POL treatment (Fig. 4B c). As shown in Fig. 4C and Fig. 4D, when autophagy was suppressed by 3-MA, POL decreased the rhodamine 123 fluorescence intensity in a time-dependent manner. Furthermore, cleavage of caspase-3 and -9 could be rescued in the presence of 10 µM z-VAD-fmk in POL-induced A549 cells (Fig. 4E). And, Sub-G_1_ cell proportion analysis and Annexin V/PI double staining were also demonstrated that caspase inhibitor z-VAD-fmk could protect A549 cells from apoptotic cell death (Fig. 4F and Fig. 4G). All these results clearly indicate that POL induced apoptosis through mitochondrial-mediated pathway in A549 cells.

### Inhibition of autophagy partially reduces POL-induced apoptosis

**Figure 5:**
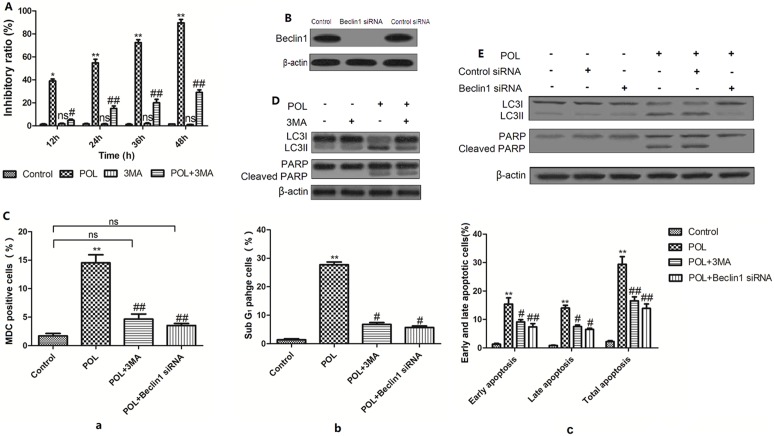
Inhibition of autophagy results in reduction of apoptosis by POL. (A) A549 cells were pretreated with 2 mM 3MA 1 h prior to the administration of 23 µg/ml POL for indicated time, and the cell inhibitory ratio was measured by MTT assay. Control group was treated with PBS. (mean±SEM, *n* = 3) (*p<0.05, **p<0.001 *vs.* Control group, ns: no significant *vs*. Control group, #p<0.05, ##p<0.001 *vs*. POL group). (B) A549 cells were transfected with control siRNA and specific Beclin1 siRNA for 24 h, the Beclin1 level was examined by Western blot analysis. (C) A549 cells were pretreated with 2 mM 3MA or transfected with specific Beclin1 siRNA prior to the administration of 23 µg/ml POL for 24 h, and the MDC fluorescent intensity (a), SubG_1_ phage cell ratio (b) and early as well as late apoptosis (c) were analyzed by flow cytometry. Control group was treated with PBS for 24 h. Data were represented as bar diagram. (mean±SEM, *n* = 3) (*p<0.05, **p<0.001 *vs.* Control group, ns: no significant *vs.* Control group, #p<0.05, ##p<0.001 *vs.* POL group). (D) Cleaved PARP and LC3-II expressions in A549 cells treated with 23 µg/ml POL for 24 h in the absence or presence of 3MA (2 mM) were measured. The representative figures were shown. (E) A549 cells were preferentially transfected with control siRNA or specific Beclin1 siRNA for 24 h before 23 µg/ml POL treatment for another 24 h, and cleavage of PARP as well as expression of LC3II were measured.

As shown in Fig. 5A, after autophagy was suppressed, POL-induced cell growth-inhibition was significantly decreased. Inhibition of autophagy by 3MA or knockdown of Beclin1 by pretreatment with Beclin1 siRNA (Fig. 5B) decreased the ratio of MDC positive cells (Fig. 5C a), indicating that autophagy was suppressed by 3MA and Beclin1 siRNA. Besides, depressing of POL-induced autophagy inhibited POL-induced apoptosis in A549 cells, which were featured by reduction of sub-G_1_ phage DNA content (Fig. 5C b), as well as early and late apoptotic portion (Fig. 5C c). Consistently, suppression of autophagy by either 3MA or Beclin1 siRNA also led to decrease in PARP cleavage and LC3-II expression in POL treated A549 cells (Fig. 5D and Fig. 5E). All these results indicated that inhibition of autophagy partially reduces POL-induced apoptosis in A549 cells.

### POL triggers apoptosis through suppressing Akt-NF-κb pathway and initiates autophagy *via* inhibiting Akt-mTOR pathway

**Figure 6:**
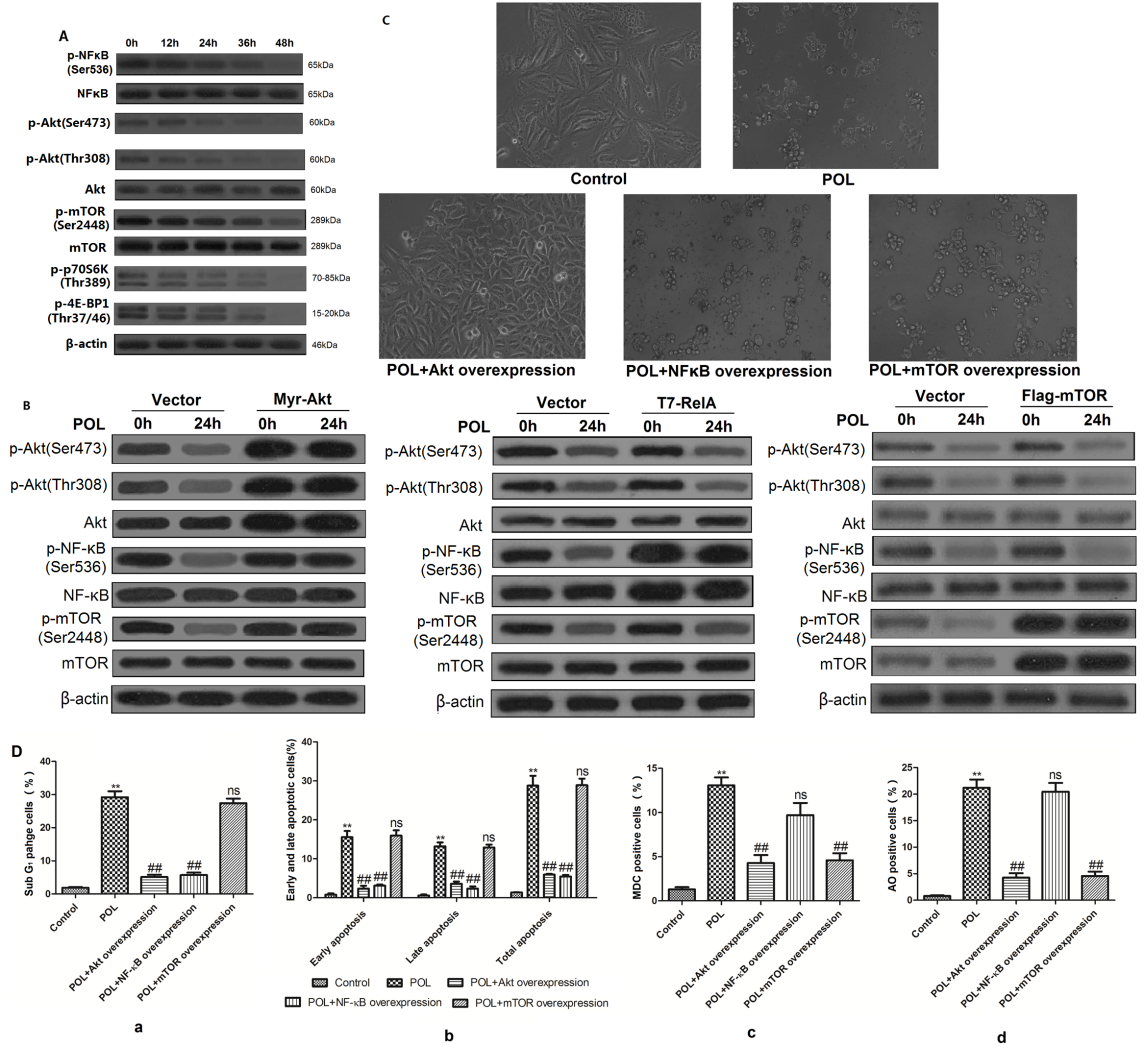
POL induces apoptosis through suppressing Akt-NF-κB pathway and autophagy via inhibiting Akt-mTOR pathway. (A) After treated with 23 µg/ml POL for indicated time, the levels of phosphorylated-Akt, Akt, phosphorylated-NF-κB (p65), NF-κB (p65), phosphorylated-mTOR, mTOR, phosphorylated-p70S6K and phosphorylated-4E-BP1 were detected. β-actin was used as an equal loading control. (B) A549 cells were transfected with empty vector, Myr-Akt (activated), T7-RelA (activated) and Flag-mTOR (activated), and treated with 23 µg/mL POL for 0 h or 24 h. The levels of phosphorylated-Akt, Akt, phosphorylated-NF-κB (p65), NF-κB (p65), phosphorylated-mTOR and mTOR were measured. β-actin was used as an equal loading control. (C) Continuous activation of Akt, NF-κB and mTOR prio to 23 µg/mL POL treatment for 24 h, the morphologic varieties were detected under phase contrast microscopy. Control group was treated with PBS for 24 h. (D) After continuous activation of Akt, NF-κB and mTOR, 23 µg/mL POL was treated with A549 cells for 24 h, the SubG_1_ phage ratio (a), early as well as late apoptosis ratio (b), MDC fluorescent intensity (c), and AVO fluorescent intensity (d) were analyzed by flow cytometry. Control group was treated with PBS for 24 h. Data were represented as bar diagram. (mean±SEM, *n* = 3). (*p<0.05, **p<0.001 *vs*. Control group, ns: no significant *vs.* POL group, #p<0.05, ##p<0.001 *vs.* POL group).

Western blot analyses showed that treatment of A549 cells with 23 µg/mL POL for indicated time resulted in decreased expressions of phosphorylated-NF-κB (p65)(Ser536), phosphorylated-Akt(Ser473), phosphorylated-Akt(Thr308), phosphorylated-mTOR(Ser2448), phosphorylated-70S6K(Thr389) and phosphorylated-4E-BP1(Thr37/46) (Fig. 6A). And the downstream substrates of mTORC1, p70S6K (70 kDa ribosomal S6 kinase) and 4E-BP1, were also efficiently dephosphorylated by POL treatment in A549 cells (Fig. 6A). In order to confirm the signaling pathway in POL-treated A549 cells, we used genetic approach to respectively over-express Akt, NF-κB and mTOR (Fig. 6B) in POL-treated A549 cells. As shown in Fig. 6B, there was strong activation of Akt, NF-κB and mTOR in transfected A549 cells compared with the vector-transfected ones, confirming the transfection efficiency. Transfection of Myr-Akt in A549 cells (over-expression of Akt) reversed POL-induced reduction of phosphorylated-NF-κB (p65)(Ser536) and phosphorylated-mTOR(Ser2448) (Fig. 6B). However, Transfection of T7-RelA (over-expression of NF-κB) or Flag-mTOR (over-expression of mTOR) did not result in any reverse of the proteins (Fig. 6B), indicating that Akt might be the upstream of NF-κB and mTOR in POL-treated A549 cells.

To further verify whether these proteins are participated in POL-induced apoptosis or autophagy, morphology detection and flow cytometry assay were applied. As shown in Fig. 6C, over-expression of Akt reversed POL-induced cell death, while over-expression of NF-κB and mTOR still partially maintained POL-induced cell death. It is indicated that Akt might be in charge of POL-induced both apoptosis and autophagy in A549 cells. Furthermore, Akt over-expression resulted in the decrease of SubG_1_ portion (Fig. 6D a), early apoptotic (Annexin V+/PI−) and late apoptotic (Annexin V+/PI+) cells (Fig. 6D b), MDC fluorescent intensity (Fig. 6D c) as well as acidic vesicular organelles (AVO) formation (Fig. 6D d). These results demonstrated that over-expression of Akt could reverse POL-induced apoptosis and autophagy simultaneously, indicating Akt protein might involve in both POL-induced apoptosis and autophagy. However, over-expression of NF-κB only resulted in suppression of apoptotic features, including significantly decreased portions of SubG_1_ (Fig. 6D a) as well as early and late apoptotic cells (Fig. 6D b). While, over-expression of mTOR only reversed POL-induced autophagy, as characterized by reduced fluorescent intensity of MDC (Fig. 6D c) and AO (Fig. 6D d). Taken together, we can conclude that POL induced apoptosis through suppressing Akt-NF-κB pathway while initiated autophagy *via* inhibiting Akt-mTOR pathway. Akt drives the switch between autophagy and apoptosis in POL-treated A549 cells.

### POL-induced molecular switch role of Akt is ROS-dependent

**Figure 7:**
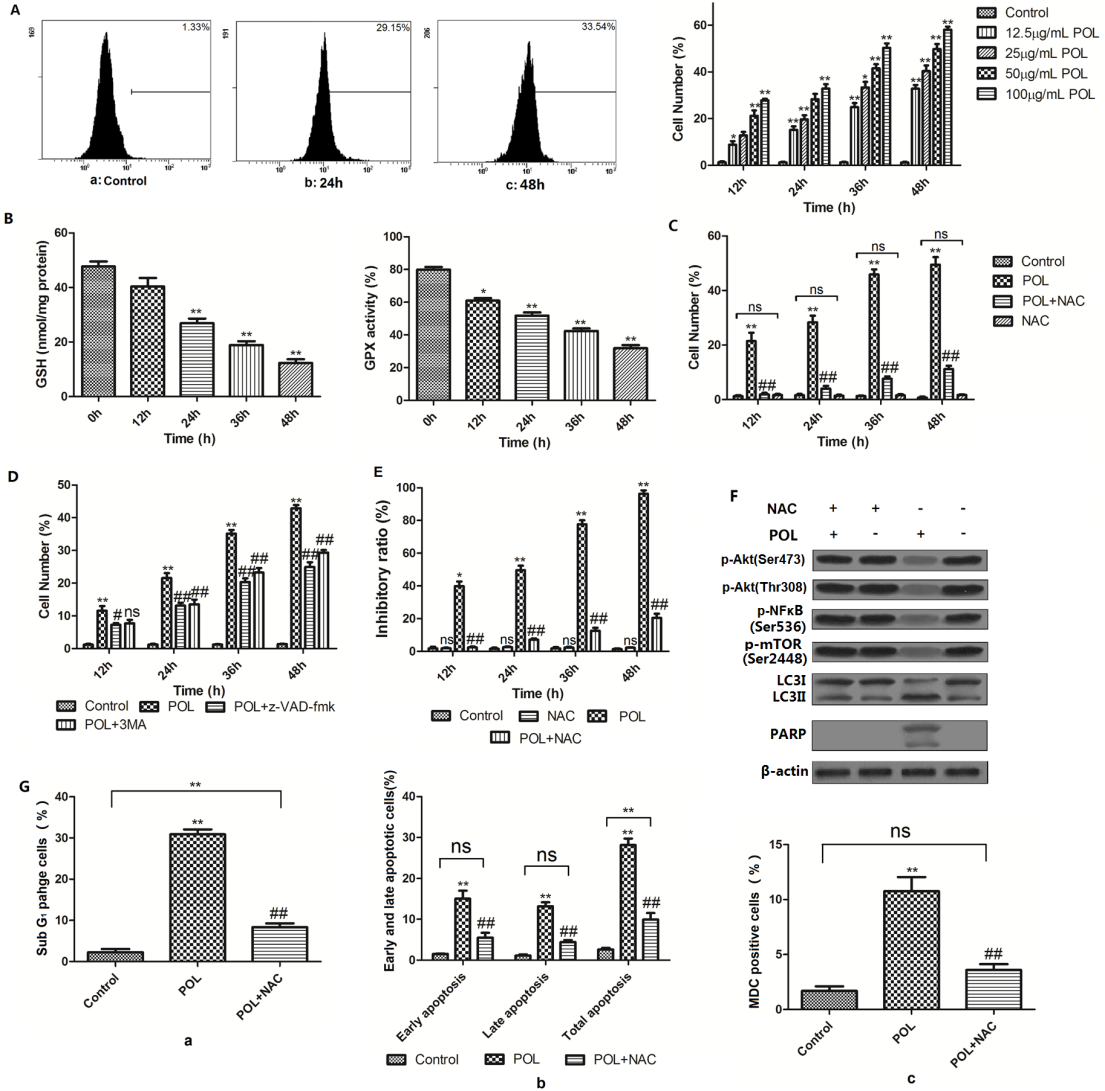
The molecular switch role of Akt was ROS-dependent. (A) Increase of cytosolic ROS levels in different concentration of POL-treated A549 cells for indicated time were detected, as shown by the enhancement of fluorescence intensity of DCF. Representative figures were shown, and all the results were represented as bar diagram. Control group was treated with PBS. (mean±SEM, *n* = 3) (*p<0.05, **p<0.001 *vs.* Control group). (B) After treatment of A549 cells with 23 µg/ml POL for different time period, the cellular levels of GSH and GPX were measured. (C) Cells were treated with 23 µg/ml POL for indicated time in the presence or absence of 4 mM NAC, and the DCF fluorescent intensity was measured by flow cytometry. Control group was treated with PBS. (mean±SEM, *n* = 3) (**p<0.001 *vs*. Control group, ##p<0.001 *vs*. POL group, ns: no significant *vs*. Control group). (D) A549 cells were pretreated with 20 µM caspase inhibitor z-VAD-fmk or 2 mM autophagic inhibitor 3MA prio to 23 µg/ml POL treatment for indicated time, and cytosolic ROS levels were detected by DCF intensity. Control group was treated with PBS. (mean±SEM, *n* = 3) (*p<0.05, **p<0.001 *vs.* Control group, #p<0.05, ##p<0.001 *vs.* POL group, ns: no significant *vs*. POL group). (E) A549 cells were pretreated with 4 mM NAC prior to the administration of 23 µg/ml POL for indicated time, and the cell inhibitory ratio was measured by MTT assay. Control group was treated with PBS. (mean±SEM, *n* = 3) (*p<0.05, **p<0.001 *vs.* Control group, #p<0.05, ##p<0.001 *vs.* POL group, ns: no significant *vs.* Control group). (F) Expressions of phosphorylated-Akt, phosphorylated-NF-κB (p65), phosphorylated-mTOR, LC3II and cleavage of PARP in A549 cells treated with 23 µg/ml POL (24 h) in the absence or presence of 4 mM NAC were measured. The representative figures were shown. β-actin was used as an equal loading control. (G) Pretreatment of A549 cells with 4 mM NAC prio to 23 µg/mL POL treatment for 24 h, the SubG_1_ phage ratio (a), early as well as late apoptosis ratio (b) and MDC fluorescent intensity (c) were analyzed by flow cytometry. Control group was treated with PBS for 24 h. Data were represented as bar diagram. (mean±SEM, *n* = 3). (*p<0.05, **p<0.001 *vs.* Control group, ns: no significant *vs.* Control group, #p<0.05, ##p<0.001 *vs.* POL group).

ROS levels were detected using ROS-detecting fluorescent dye DCFH-DA. It is demonstrated that ROS accumulation was enhanced in POL-induced A549 cells in a dose- and time-dependent manner (Fig. 7A). Furthermore, incubation of A549 cells with 23 µg/mL POL led to the reduction of GSH content and GPX enzyme activity in a time-dependent manner, suggesting that POL abrogated the GSH antioxidant system and finally resulted in massive ROS accumulation in A549 cells (Fig. 7B). And, this 23 µg/mL POL-induced increase of ROS could be inhibited by pretreatment with the ROS scavenger, NAC (Fig. 7C). Moreover, this increase could also be inhibited by pretreatment with apoptosis inhibitor z-VAD-fmk or autophagy inhibitor 3-MA (Fig. 7D). These results demonstrated that ROS might be implicated in POL-induced both apoptosis and autophagy.

Complement with these findings, ROS inhibitor NAC could also reverse POL-induced cytotoxicity (Fig. 7E), and suppress POL-induced reduction of phosphorylated-Akt, phosphorylated-NF-κB and phosphorylated-mTOR levels, the cleavage of caspase 3, and the accumulation of LC3II (Fig. 7F). From mentioned-above results we can inferred that ROS was involved in POL-induced inhibition of Akt expression, and might therefore mediate both apoptosis and autophagy.

In order to further detect whether POL-induced ROS generation was associated with POL-induced apoptosis and autophagy, SubG_1_ portion (Fig. 7G a), early apoptotic (Annexin V+/PI−) and late apoptotic (Annexin V+/PI+) cell portion (Fig. 7G b), MDC fluorescent intensity (Fig. 7G c) were determined by flow cytometry. As expected, POL-induced ROS generation was not only implicated in POL-induced apoptosis, but also in POL-induced autophagy.

## Discussion

In the present study, POL bears different anti-neoplastic effects toward different cancer cells, of which POL was more sensitive to non-small cell lung cancer A549 cells with IC_50_ value at 23 µg/mL (24 h). However, POL exhibited neither apoptosis- nor autophagy-inducing effects toward normal HELF cells, suggesting POL bears a greater susceptibility to the malignant cells. These results indicated that POL bears the potential to be used in the treatment of different types of cancer, but further *in vivo* xenograft study is still needed to evaluate its anti-neoplastic effects.

Plant lectins bear affinity toward different carbohydrates, herein, three types of mannoses completely suppressed growth inhibitory effect of POL. These results suggested that there may be a close link between the hemagglutinating activity and anti-proliferative activity of POL. Like other lectins, POL might exert its death-inducing property through binding with high content of mannose- or glucose-containing moieties on the cell membrane receptors [18].

With the increasing culturing time, the percentages of early as well as late apoptotic cells were enhanced, while the percentage of alive cells was decreased in POL-treated A549 cells. POL treatment resulted in activation of caspase3/9, release of cytochrome *c*, reduction of mitochondrial membrane potential, up-regulation of pro-apoptotic Bax and Bid, down-regulation of anti-apoptotic Bcl-2 and Bcl-X_L_, as well as cleavage of PARP, all of which are the features of caspase- and mitochondrial-dependent apoptosis. In addition to apoptosis, POL also induced autophagy in A549 cells, as indicated by a time-dependent increase in the formation of acidic vesicular organelles (AVO) in macrophages, enhancement of Beclin1 expression and transformation of LC3I to LC3II.

Recently, a viewpoint has been widely accepted that there might exist positive or negative correlation between apoptosis and autophagy. Autophagy might promote or regulate apoptotic cell death, but under certain circumstances, autophagy could be initiated only when apoptosis was restrained [19]. Unlike apoptosis, autophagy plays not only pro-survival mechanism against nutrient shortage, but also paradoxically acts as a route to cancer cell death [20]. However, whether cells undergo survival or death mainly rely on cell type, drugs as well as concentration and duration. Herein, we reported that inhibition of autophagy could reduce POL-induced apoptosis. Suppression of POL-induced autophagy resulted in decreased growth inhibitory ratio, and also deprive of autophagy by either 3MA or Beclin1 siRNA led to decrease in the ratio of apoptotic cells and the cleavage of PARP in POL-induced A549 cells.

Cancerous growth is one of the most difficult diseases to cure, and nowadays researchers accepted that targeting only one form of PCD does not generally produce quantifiable improvement. And also, these two kinds of PCD might share some common factors [21], suggesting the existence of ‘molecular switch’ between apoptosis and autophagy [22]. Herein, POL was further demonstrated to induce decreased expressions of phosphorylated-NF-κB (p65), phosphorylated-Akt(Ser473/Thr308), phosphorylated-mTOR, phosphorylated-70S6K and phosphorylated-4E-BP1. Through consecutive activation of NF-κB, Akt or mTOR by genetic approach, we demonstrated that POL induced apoptosis *via* inhibiting Akt-NF-κB pathway while triggered autophagy *via* suppressing Akt-mTOR pathway. Also, after POL treatment of A549 cells, the levels of ROS were enhanced, and inhibition of ROS through inhibitor NAC resulted in the decrement of phosphorylated-Akt (Ser473/Thr308) expressions as well as portion of apoptotic and autophagic cells.

Therefore, we could infer that Akt might exert the ‘molecular switch’ role in regulating between POL-induced apoptosis and autophagy. After POL treatment, cells might undergo apoptotic cell death if Akt-NF-κB pathway was inhibited, while cells might also exhibit pro-death autophagy when Akt-mTOR signaling was suppressed. Akt is frequently activated in non-small cell lung cancer cells, and usually, nuclear factor kappa-light-chain-enhancer of activated B cells (NF-κB) acts as downstream of Akt [23]. Activation of NF-κB is dependent on the phosphorylation of IκB kinase (IKKα), which is under the control of Akt kinase [24]. Akt-mTOR pathway is essential in the regulation of autophagic cell death, and usually, mTOR interacting with Raptor forms rapamycin-sensitive TORC1 complex, which controls phosphorylation of p70S6K (70 kDa ribosomal S6 kinase) [25]. Also, mTOR binding with Rictor forms rapamycin-insensitive complex, TORC2, which in turn modulates phosphorylation of Akt [26]. After POL treatment, ROS was gradually accumulated in A549 cells, and ROS is in charge of the dephosphorylation of Akt, inferring that ROS might regulate the molecular switch role of Akt. Intracellular ROS are exquisitely balanced by nonenzymatic antioxidants (like glutathione (GSH)) and antioxidant enzymes, and if the balance toward the oxidant side, causes damage to biomolecules such as DNA, proteins, and lipids [27]. Herein, we demonstrated that nonenzymatic antioxidants GSH and its partner GPX were decreased with culturing time, indicating that POL might abrogate the GSH antioxidant system and result in massive ROS accumulation in A549 cells.

Numerous plant lectins were verified to bear apoptosis- and autophagy-inducing effects, like *polygonatm cyrtonema* lectin (PCL) [10], [11] and concanavalin A (ConA) [27], [28]. PCL simultaneously induced apoptosis and autophagy in human melanoma A375 cells *via* mitochondrial-mediated ROS-p38-p53 pathway [10], and also initiated apoptosis and autophagy *via* blocking Ras-Raf and PI3K-Akt signaling pathways in murine fibrosarcoma L929 cells [11]. Besides, legume lectin, ConA, could induce caspase-dependent apoptosis in human melanoma A375 cells [28], as well as BNIP3-mediated mitochondrial autophagy [29] in HepG_2_ cells.

**Figure 8:**
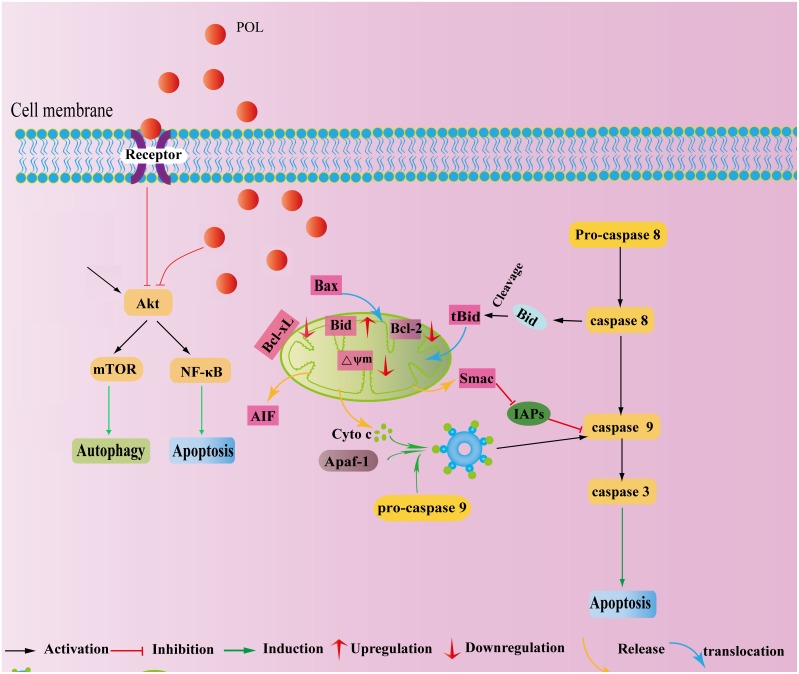
The cartoon illustrating molecular basis of POL-induced apoptosis and autophagy.

However, the regulation of tumor cell death might be considered as a ‘dynamic balance’ between apoptosis and autophagy [30]. Still, there exist a complex and not fully defined relationship between them [31], and the exact ‘molecular switch’ role of Akt needs to be further verified. Whether there exist precedence between POL-induced apoptosis and autophagy? How did Akt protein decide between POL-induced apoptosis and autophagy? Why POL-induced cell death resulted in ROS accumulation? How did ROS exert regulatory effect on Akt expression and POL-induced cell death? All above-mentioned questions are still hanging in doubt, and more strict and deep experiments should be further performed. And we have summarized all the pathways in Fig. 8.

In summary, all above-mentioned findings would provide new evidence for further exploring more PCD mechanisms of GNA-related lectins in cancer research. With the complex molecular mechanisms of GNA-related lectin-induced PCD becoming better elucidated, there is no doubt that new therapeutic strategies would be developed into targeting apoptotic and autophagic cell death pathways for cancer therapeutics.

## References

[1] Sharon N, Lis H (1989) Lectins as cell recognition molecules. Science 246: 227–234. 2552581 10.1126/science.2552581

[2] Van Damme EJ, Smith DF, Cummings R, Peumans WJ (2011) Glycan arrays to decipher the specificity of plant lectins. Adv Exp Med Biol 705: 757–767. 21618139 10.1007/978-1-4419-7877-6_39

[3] Fu LL, Zhou CC, Yao S, Yu JY, Liu B, et al (2011) Plant lectins: targeting programmed cell death pathways as antitumor agents. Int J Biochem Cell Biol 43: 1442–1449. 21798364 10.1016/j.biocel.2011.07.004

[4] Li CY, Luo P, Liu JJ, Wang EQ, Li WW, et al (2011) Recombinant expression of *Polygonatum cyrtonema* lectin with anti-viral, apoptosis-inducing activities and preliminary crystallization. Process Biochem 46: 533–542.

[5] Mathew R, Karantza-Wadsworth V, White E (2007) Role of autophagy in cancer. Nat Rev Cancer 7: 961–967. 17972889 10.1038/nrc2254 PMC2866167

[6] Shi Z, Li CY, Zhao S, Yu Y, An N, et al (2013) A systems biology analysis of autophagy in cancer therapy. Cancer Lett 337: 149–160. 23791881 10.1016/j.canlet.2013.06.004

[7] Li CY, Meng L, Liu B, Bao JK (2009) *Galanthus nivalis* Agglutinin (GNA)-Related Lectins: Traditional Proteins, Burgeoning Drugs? Curr Chem Biol 3: 324–333.

[8] Yang Y, Xu HL, Zhang ZT, Liu JJ, Li WW, et al (2011) Characterization, molecular cloning, and *in silico* analysis of a novel mannose-binding lectin from *Polygonatum odoratum* (*Mill.*) with anti-HSV-II and apoptosis-inducing activities. Phytomedicine 18: 748–755. 21146383 10.1016/j.phymed.2010.11.001

[9] Liu B, Zhang B, Min MW, Bian HJ, Chen LF, et al (2009) Induction of apoptosis by *Polygonatum odoratum* lectin and its molecular mechanisms in murine fibrosarcoma L929 cells. Biochim Biophys Acta 1790: 840–844. 19414060 10.1016/j.bbagen.2009.04.020

[10] Liu B, Cheng Y, Zhang B, Bian HJ, Bao JK (2009) Polygonatum cyrtonema lectin induces apoptosis and autophagy in human melanoma A375 cells through a mitochondria-mediated ROS–p38–p53 pathway. Cancer Lett 275: 54–60. 19010591 10.1016/j.canlet.2008.09.042

[11] Liu B, Wu JM, Li J, Liu JJ, Li WW, et al (2010) *Polygonatum cyrtonema* lectin induces murine fibrosarcoma L929 cell apoptosis and autophagy *via* blocking Ras–Raf and PI3K–Akt signaling pathways. Biochimie 92: 1934–1938. 20713122 10.1016/j.biochi.2010.08.009

[12] Xu HL, Li CY, He XM, Niu KQ, Peng H, et al (2012) Molecular modeling, docking and dynamics simulations of GNA-related lectins for potential prevention of influenza virus (H1N1). J Mol Model 18: 27–37. 21445708 10.1007/s00894-011-1022-7

[13] Shi Z, Wang ZJ, Xu HL, Tian Y, Li X, et al (2013) Modeling, docking and dynamics simulations of a non-specific lipid transfer protein from *Peganum harmala L* . Comput Biol Chem. 47: 56–65. 23891721 10.1016/j.compbiolchem.2013.07.001

[14] Cheng Y, Qiu F, Tashiro SI, Onodera S, Ikejima T (2008) ERK and JNK mediate TNFα-induced p53 activation in apoptotic and autophagic L929 cell death. Biochem Biophys Res Commun 376: 483–488. 18796294 10.1016/j.bbrc.2008.09.018

[15] Li CY, Wang Y, Wang HL, Shi Z, An N, et al (2013) Molecular mechanisms of *Lycoris aurea* agglutinin-induced apoptosis and G_2_/M cell cycle arrest in human lung adenocarcinoma A549 cells, both *in vitro* and *in vivo* . Cell Prolif 46: 272–282. 23692086 10.1111/cpr.12034 PMC6495545

[16] Wang Y, Liu YD, Wang HL, Li CY, Qi P, et al (2012) *Agaricus bisporus* lectins mediates islet β-cell proliferation through regulation of cell cycle proteins. Exp Bio Med 237: 287–296. 10.1258/ebm.2011.011251 22393165

[17] Wang Y, Wang HL, Liu YD, Li CY, Qi P, et al (2012) Antihyperglycemic effect of ginsenoside Rh2 by inducing islet β-cell regeneration in mice. Horm Metab Res 44: 33–40. 22205570 10.1055/s-0031-1295416

[18] Lei HY, Chang CP (2007) Induction of autophagy by concanavalin A and its application in anti-tumor therapy. Autophagy 3: 402–404. 17471013 10.4161/auto.4280

[19] Thorburn A (2008) Apoptosis and autophagy: regulatory connections between two supposedly different processes. Apoptosis 13: 1–9. 17990121 10.1007/s10495-007-0154-9 PMC2601595

[20] Li CY, Wang EQ, Cheng Y, Bao JK (2011) Oridonin: An active diterpenoid targeting cell cycle arrest, apoptotic and autophagic pathways for cancer therapeutics. Int J Biochem Cell B 43: 701–704. 10.1016/j.biocel.2011.01.020 21295154

[21] Edinger AL, Thompson CB (2004) Death by design: apoptosis, necrosis and autophagy. Curr Opin Cell Biol 16: 663–669. 15530778 10.1016/j.ceb.2004.09.011

[22] Cheng Y, Ren XC, Hait WN, Yang JM (2013) Therapeutic targeting of autophagy in disease: biology and pharmacology. Pharmacol Rev 65: 1162–1197. 23943849 10.1124/pr.112.007120 PMC3799234

[23] Lee YC, Lin HH, Hsu CH, Wang CJ, Chiang TA, et al (2010) Inhibitory effects of andrographolide on migration and invasion in human non-small cell lung cancer A549 cells via down-regulation of PI3K/Akt signaling pathway. Eur J Pharmacol 632: 23–32. 20097193 10.1016/j.ejphar.2010.01.009

[24] Sun ZJ, Chen G, Hu X, Zhang W, Liu Y, et al (2010) Activation of PI3K/Akt/IKK-α/NF-κB signaling pathway is required for the apoptosis-evasion in human salivary adenoid cystic carcinoma: its inhibition by quercetin. Apoptosis 15: 850–863. 20386985 10.1007/s10495-010-0497-5

[25] Hennessy BT, Smith DL, Ram PT, Lu Y, Mills GB (2005) Exploiting the PI3K/AKT pathway for cancer drug discovery. Nat Rev Drug Discov 4: 988–1004. 16341064 10.1038/nrd1902

[26] Ciuffreda L, Di Sanza C, Incani UC, Milella M (2010) The mTOR pathway: a new target in cancer therapy. Curr Cancer Drug Targets 10: 484–495. 20384580 10.2174/156800910791517172

[27] Liu B, Chen Y, St Clair DK (2008) ROS and p53: A versatile partnership. Free Radic Biol Med 44: 1529–35. 18275858 10.1016/j.freeradbiomed.2008.01.011 PMC2359898

[28] Liu B, Li CY, Bian HJ, Min MW, Chen LF, et al (2009) Antiproliferative activity and apoptosis-inducing mechanism of Concanavalin A on human melanoma A375 cells. Arch Biochem Biophys 482: 1–6. 19111670 10.1016/j.abb.2008.12.003

[29] Chang CP, Yang MC, Liu HS, Lin YS, Lei HY (2007) Concanavalin A induces autophagy in hepatoma cells and has a therapeutic effect in a murine in situ hepatoma model. Hepatology 45: 286–296. 17256764 10.1002/hep.21509

[30] Kessel D, Reiners JJ Jr (2007) Initiation of apoptosis and autophagy by the Bcl-2 antagonist HA14-1. Cancer Lett 249: 294–299. 17055152 10.1016/j.canlet.2006.09.009 PMC1924967

[31] Choi KS (2012) Autophagy and cancer, Exp Mol Med. 44: 109–120. 22257886 10.3858/emm.2012.44.2.033 PMC3296807

